# Anti-*N*-methyl-D-aspartate receptor encephalitis: a newly recognized inflammatory brain disease in children

**DOI:** 10.1186/1546-0096-10-S1-A82

**Published:** 2012-07-13

**Authors:** Nadia JC Luca, Tassalapa Daengsuwan, Josep Dalmau, Kevin Jones, Gabrielle deVeber, Jeff Kobayashi, Ronald M Laxer, Susanne M Benseler

**Affiliations:** 1 Hospital for Sick Children, Toronto, ON, Canada; 2 University of Pennsylvania, Philadelphia, PA, USA

## Purpose

Inflammatory brain disease causes severe psychiatric and neurological deficits in previously healthy children. The aim of this study was to report characteristic clinical features and outcomes of children diagnosed with Anti-*N*-methyl-D-aspartate receptor (NMDAR) encephalitis, a newly recognized anti-neuronal antibody-mediated inflammatory brain disease, seen by the Rheumatology service over an 18 month period.

## Methods

Over an 18-month period (July 2009-Dec 2010), consecutive children presenting with newly acquired psychiatric and/or neurologic deficits consistent with anti-NMDAR encephalitis and evidence of central nervous system (CNS) inflammation were screened. Serum and cerebrospinal fluid (CSF) samples were obtained and sent for anti-NMDAR antibody testing. Children were included in the study if they had confirmatory evidence of these antibodies in the serum and/or CSF. Details of clinical presentation and results of investigations, including inflammatory markers, CSF studies and neuroimaging, were documented. Type and duration of treatment and outcomes at last follow-up were evaluated.

## Results

Over the study period a total of 18 children presented with clinical features compatible with anti-NMDAR encephalitis including psychiatric manifestations, seizures and/or movement disorders. Four children (22%) had positive anti-NMDAR antibodies and were diagnosed with anti-NMDAR encephalitis. These included one male and three females, with a median age of 12.8 years (range 3-16 years). The remaining 14 children were subsequently diagnosed with 1) primary CNS vasculitis (4); 2) post-infectious inflammatory brain disease (2); 3) channelopathy (1); 4) reversible splenial lesions syndrome (1); 5) epilepsy (1); and 6) inflammatory brain disease NYD (5). All children with anti-NMDAR encephalitis presented with neuropsychiatric deficits including, seizures, speech disorder, sleep disturbance, and fluctuating level of consciousness. The three older patients also had prominent psychiatric features, while the younger child had significant autonomic instability and prominent involuntary movement disorder. None had an underlying tumor. Immunosuppressive therapy with intravenous immunoglobulin, steroids and/or rituximab, resulted in near or complete recovery; however, two of the patients had early relapse requiring re-treatment.

## Conclusion

Anti-NMDAR encephalitis is an important, reversible cause of neuropsychiatric deficits in children that must be included in the differential diagnosis of CNS vasculitis and other inflammatory brain diseases. Early diagnosis and treatment are essential for neurologic recovery.

## Disclosure

Nadia J.C. Luca: None; Tassalapa Daengsuwan: None; Josep Dalmau: Euroimmun, 2, 9, Individual patent, 2, 9; Kevin Jones: None; Gabrielle deVeber: None; Jeff Kobayashi: None; Ronald M. Laxer: None; Susanne M. Benseler: None.

